# Reducing resistance to treatment, through group intervention, improves clinical measurements in patients with type 2 diabetes

**DOI:** 10.1186/1472-6823-13-61

**Published:** 2013-12-28

**Authors:** Liora Valinsky, Moshe Mishali, Ronit Endevelt, Rachel Preiss, Keren Dopelt, Anthony D Heymann

**Affiliations:** 1 Department of Clinical Quality, Meuhedet Health Care, 124 Ibn-Gvirol St, Tel Aviv, Israel; 2 Department of Preventive Medicine, Maccabi Heathcare Services, Tel Aviv, Israel; 3 School of public health Haifa University Carmel, Haifa, Israel; 4 Department of Health Systems Management, Ben Gurion University of the Negev, Beer Sheva, Israel; 5 Department of Psychology, Tel Aviv University, Tel Aviv, Israel; 6 Sackler Faculty of Medicine, Tel Aviv University, Tel Aviv, Israel

**Keywords:** Empowerment, Diabetes mellitus, Self-help groups, Resistance to treatment, Family support

## Abstract

**Background:**

Studies have shown that group Therapeutic Patient Education (TPE) may empower patients with type 2 diabetes to better manage their disease. The mechanism of these interventions is not fully understood. A reduction in resistance to treatment may explain the mechanism by which TPE empowers participants to improve self-management. *The Objective* of this study was to examine the effectiveness of diabetes groups in reducing resistance to treatment and the association between reduced resistance and better management of the disease.

**Methods:**

In a program evaluation study, we administered validated questionnaires to measure resistance to treatment (RTQ) in 3 time periods: before the intervention (T1), immediately after the intervention (T2) and six months later (T3). Clinical measures (HbA1C, blood pressure, HDL, LDL and total cholesterol, Triglycerides and BMI) were retrieved from Maccabi Healthcare Services computerized systems, for T1;T2 and a year post intervention (T3). Linear mixed models were used adjusting for age, gender, social support and family status.

**Results:**

*157; 156 and 106* TPE participants completed the RTQ in T1; T2 and T3 respectively. HbA1C and systolic and diastolic blood pressure were significantly reduced in the group which achieved a reduction in three out of the five RTQ components. For the other clinical measurements no significant changes were observed.

**Conclusion:**

Our findings suggest that reducing resistance to treatment, through an educational program for patients with diabetes, is associated with a better disease control. Identifying patients with higher resistance to treatment, and including components that reduce resistance in patient education programs, have the potential to increase the effectiveness of these programs.

## Background

It is well recognized that adoption of self-management skills by patients with diabetes is necessary to enable them to manage their illness [1,2]. The British National Institute for Clinical Excellence (NICE), have suggested that the delivery of a structured self-management education program for people with diabetes, is a cornerstone of the management of this chronic condition [3,4]. This principle is now accepted as being part of the care delivered to people with diabetes.

Diabetes is an extremely expensive disease for health systems. The complications, which are mainly due to treatment failures, are caused by poor self management of the disease, rather than by poor organizational resource investments. For patients to be able to manage their disease in a reasonable manner, they need to be empowered in several dimensions: physically (access), cognitively (skills) and emotionally (self-efficacy, health beliefs, readiness for change). Any intervention designed to improve patient adherence to treatment through self empowerment, needs to address all of these aspects of care. Furthermore, participation in an empowerment-based diabetes self-management support intervention may have a positive and enduring effect on self-care behaviors and on metabolic measurements [5].

Resistance to treatment, has been identified as a reliable instrument to measure the degree to which patients are able to accept and cope with different aspect of diabetes management, including: psychological acceptance, health care team acceptance, emotional acceptance and overall acceptance of the diagnosis and its everyday implications. Additionally, resistance to treatment has been found to be associated with self-efficacy and readiness to change, and is therefore a reasonable surrogate for the measurement of patient empowerment.

Many of the interventions relating to patient behavior, are conducted within a theoretical framework, such as the Health belief Model [6] or the Trans Theoretical Model (TTM) [7-9]. However, when developing an intervention within constrained resources, it is important to develop interventions that are not only based on behavioral models, but that are based on changing patient attitudes, beliefs and behavior in a measurable manner. Resistance to treatment as measured by the RTQ can be extremely useful in this, and can serve as a model for the design of interventions such as diabetes education groups. Additionally, it can serve as a tool for a better recruitment of targeted participants (individuals that will most likely to gain from these kind of interventions) and as an evaluation tool (to adequately measure the improvement the group achieved).

Although Therapeutic Patient Education (TPE), for people with diabetes can be undertaken on an individual level, by a healthcare provider such as a nurse [10], dietician or a physician, by peer groups [11] or electronic remote learning [12], NICE has recommended that diabetes education should be provided by an appropriately qualified multidisciplinary teams to groups of people with the disease. Over time, the emphasis in these educational programs has shifted from a didactic approach to patient empowerment approaches [10] and it is clear now that programs incorporating behavioral and psychosocial strategies with or without ethnic and cultural adaptation have demonstrated improved outcomes [11-13]. It was suggested by previous studies, that patients with type 2 diabetes who have participated in TPE programs based on self-management strategies improved their diabetes control: fasting blood glucose and glycosylated hemoglobin(HbA1C) and their knowledge about the disease in the short (four to six months) and longer-term (12 to 14 months), whilst also reduced the need for diabetes medication. There is also some evidence that group-based education programs may lead to reduced blood pressure and body weight, and to the increase of self-empowerment, quality of life, self-management skills and treatment satisfaction. However, only a small number of studies evaluated those outcomes [11].

Maccabi Healthcare Services (MHS) is the second largest Health Maintenance Organization (HMO) in Israel, providing primary care to 1.9 million residents nationwide through five administrative and geographical regions. Physicians are employed in independent practices, and members have free choice in regard to their own care-givers. Among MHS beneficiaries, 104,000 are patients with diabetes and the disease has become one of the major chronic diseases with a professional and economic burden.

Based on the assumption that patient empowerment groups have the potential to improve diabetes self-management MHS, for many years, encourages diabetic patients to participate in these TPEs. To date almost 8,000 Patients have participated in these groups, but other than an evaluation of patient satisfaction, no examination of the clinical impact was performed.

*The Objective* of this study was to examine the effectiveness of diabetes groups in reducing resistance to treatment and the association between reduced resistance and better management of the disease.

## Methods

### The intervention

Throughout the country we offer structured group interventions with a very modest co-payment, to empower patients by providing knowledge, tools and support. The empowerment model is based on the assumption that to improve their health, people must bring about changes not only in their personal behavior but also in their social situations and in the environment that influences their lives. This empowerment model has evolved out of the realization that patients cannot be forced to follow a lifestyle dictated by others [1].

These intervention groups are run by a specially educated team consisting of a diabetes nurse, a dietician and a social worker trained in psychosocial counseling. The intervention comprises of eight meetings over a period of two months and spouses are invited to participate. *Subjects on the agenda are*: resistance to treatment; knowledge about the disease; medications; nutrition, motivation for a change and coping with the disease demands in different social environments.

### Setting

The evaluation was performed between September 2007 and September 2008, in three districts, by MHS’ research team.

### Study population and recruitment

All participants in the all TPE groups in the selected districts between September 2007 and September 2008 were invited to take part in the study. They were all contacted in advance by telephone, to explain the aims of the study and to ask for their consent. Those who agreed were asked to arrive early to the first group meeting to sign an informed consent form and fill in the first questionnaire.

### Data collection

#### Questionnaire

We used a validated RTQ questionnaire [13] to measure resistance to treatment. The RTQ is a four theme, 40 item questionnaire to measure the core reasons for non-adherence with treatment: 1: Dissatisfaction with treatment or team (Therapy); 2: Emotional reasons (Emotions); 3: specific problems or constraints (Difficulties) and 4: factors connected to despair and failure (Despair). Scores vary between 1 (very low resistance) and 5 (very high resistance). In addition, the questionnaire includes items regarding family status; social support availability; level of education; age and gender.

#### Clinical measurement

In order to measure the degree of diabetes control, the following clinical measurements were included: HbA1C; diastolic and systolic blood pressure; HDL and LDL cholesterol; Triglycerides and Body Mass Index (BMI),. These variables were retrieved from MHS’ computerized records.

#### Time periods

**Figure 1:**
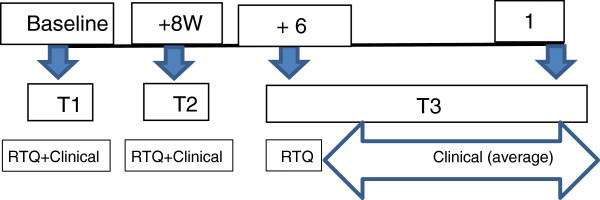
Study time periods.

Questionnaires were administered at three points: at recruitment (baseline T1); eight weeks later (at the last meeting of the intervention) (T2); and six months later (T3). Clinical measurements were obtained at T1 and T2, and one year post intervention (T3) (Figure 1).

### Data analysis

#### Study groups

For each core of the RTQ separately, participants were divided into two groups: those who achieved reduction at T2 (delta between T2 and T1 <0 - group 1) and those who failed to achieve reduction (delta between T2 and T1 = >0 - group 0).

#### Clinical measurements

Clinical results were collected for T1 and T2 at precise dates (at recruitment and at the end of the intervention). For T3 we documented routinely recorded measurements over the whole year post intervention. For this time point (T3) we calculated a 12 months average for each type of variable.

#### Statistical techniques

For each RTQ core subject separately, we analyzed the demographic variables between the study groups using T-test and Chi Square techniques, as required.

Since we had an unbalanced design of repeated measures, we applied mixed linear models. The dependent variables were the clinical measurements. The independent fixed factors were time of measurement (T1;T2 andT3) and ‘change in resistance’ groups. Subjects were entered into the model as a random factor (since we had repeated measures for each subject). For each dependent variable, we established two models. In the first model, we included two main effects: time and group in order to assess whether the two groups differ in baseline measurements. In the second model, we entered an interaction between group and time (group*time) in order to obtain the estimate of the change in the dependent variable along time in each group. All models were adjusted according to the univariate analysis (Table 1).

**Table 1:** Personal characteristics by study groups

**Variable**	**Despair**			**Emotions**		
	**Group 1**	**Group 0**	**P Value**	**Group 1**	**Group 0**	***P value***
Mean age	58.6(9.5)	56.8(9.9)	0.307	55.39(9.6)	60.3(9.2)	*0.009*
% Females	45.9	58.2	0.169	52.1	50.0	*0.817*
% <12y educ.	38.6	60.4	0.017	45.1	51.9	*0.452*
% Married	71.0	86.8	0.037	75.7	80.8	*0.506*
Mean social support	0.56(0.7)	0.76(0.7)	0.109	0.64(0.6)	0.64(0.7)	*0.997*
	**Difficulty**			**Therapy**		
	**Group 1**	**Group 0**	**P value**	**Group 1**	**Group 0**	***P value***
Mean age	57.8(9.8)	57.8(9.6)	0.972	56.8(9.9)	59.3(9.3)	*0.151*
% Females	54.3	47.5	0.440	50.0	53.1	*0.736*
% <12y educ.	45.5	50.9	0.548	42.1	57.4	*0.098*
% Married	70.1	87.3	0.023	80.0	74.5	*0.474*
Mean social support	0.59(0.6)	0.71(0.7)	0.332	0.68(0.7)	0.58(0.6)	*0.452*
	**Total resistance**					
	**Group 1**	**Group 0**	***P value***			
Mean age	57.5(9.9)	58.5(9.2)	*0.588*			
% Females	51.7	50.0	*0.859*			
% <12y educ.	44.7	55.3	*0.279*			
% Married	76.2	81.6	*0.507*			
Mean social support	0.66(0.6)	0.60(0.6)	*0.682*			

Note: Group 1:achieved reduction at T2 (delta between T2 and T1 <0); group 0: those who failed to achieve reduction (delta between T2 and T1 = >0).

#### Ethics

The intervention and the evaluation study were both approved by the local ethics committee.

## Results

Diabetes education groups are conducted routinely throughout the country. During the study period, 26 educational groups were offered to patients in the selected regions. Of *210* participants in these groups, *157* (75%) completed RTQ and had clinical data at T1; 156 (74%) at T2 and 106 (50%) at T3. Calculations of sample size revealed sufficient statistical power for 5% and 50% for Alpha and Beta errors respectively.

No statistically significant differences were found between total resistance study groups at baseline (T1); T2 and T3 (Table 2).

**Table 2:** **Clinical measurements at T1 (baseline); T2 and T3 for the two study groups**^**†**^

**Time**	**Group 1**			**Group 0**			**P value**
	**N**	**Mean**	**SD**	**N**	**Mean**	**SD**	
**HbA1C**							
T1	89	7.5	1.8	41	7.1	1.7	0.210
T2	92	7.1	1.4	38	6.9	1.26	0.461
T3	58	7.0	1.2	26	7.1	1.4	0.816
**HDL**							
T1	90	45.5	11.3	40	47.3	13.5	0.447
T2	91	45.0	11.1	39	47.7	12.9	0.265
T3	52	43.6	9.5	26	47.9	12.8	0.138
**LDL**							
T1	90	93.4	29.3	40	96.8	29.0	0.538
T2	91	91.8	32.6	39	92.1	38.2	0.965
T3	52	94.5	32.0	26	92.8	34.7	0.842
**TG**							
T1	90	155.4	70.3	40	162.5	89.5	0.657
T2	91	154.5	70.7	39	176.5	142.5	0.364
T3	53	180.1	97.8	26	190.0	144.8	0.754
**BPS**							
T1	90	132.0	16.9	41	131.0	17.2	0.769
T2	92	130.0	15.2	38	126.6	15.4	0.259
T3	55	129.3	13.6	23	134.1	16.3	0.223
**BPD**							
T1	90	79.0	10.0	41	79.0	9.6	0.992
T2	92	77.6	8.9	38	76.5	9.4	0.517
T3	55	74.7	8.3	23	75.7	10.2	0.675
**BMI**							
T1	88	30.7	5.2	41	29.8	4.9	0.362
T2	89	31.2	6.3	38	30.2	4.8	0.349
T3	58	29.9	4.7	23	29.4	4.5	0.630

SD = Standard Deviation; † Study groups = **Total Resistance**: delta between T2 and T1 <0 = group 1; delta between T2 and T1 = >0 - group 0

Level of education was statistically different between study groups in the Despair core; mean age in the Emotions core and family status in the Despair and difficulty cores (Table 1). Study groups were found to be statistically significant different in all resistance cores at baseline, meaning group 1 scores were higher than those of group 0 (Table 3). The results of the linear mixed models revealed no significant differences between the study groups at baseline in all clinical measurements (Table 4). HbA1C was significantly reduced in group 1 between time periods, in the Despair; Difficulty and Emotion cores while group 0 reduced HbA1C in the Therapy core and Total Resistance. Diastolic blood pressure was reduced in group 1 in all RTQ cores however, systolic blood pressure was reduced in group 1 only in Difficulty and therapy cores. For other clinical measurements no significant changes were observed (Table 4).

**Table 3:** Resistance scores at baseline (1very low; 5 very high) by study groups

**RTQ Realm**	**Group 1**	**Group 0**	**P value**
Dissatisfaction with treatment or team (Therapy)	2.1	1.8	0.002
Emotional reasons (Emotions)	2.1	1.8	0.007
Specific problems (Difficulty)	2.7	2.4	0.003
Despair and failure (Despair)	2.1	1.7	0.005
Total resistance to treatment (total resistance)	2.2	2.0	0.254

Note: Group 1:achieved reduction at T2 (delta between T2 and T1 <0); group 0: those who failed to achieve reduction (delta between T2 and T1 = >0).

**Table 4:** Results of the linear mixed models for resistance to treatment by study groups and time

	**Total resistance**		**Despair**		**Difficulty**		**Therapy**		**Emotions**	
**HbA1C/P value at baseline**^**α**^	0.101		0.968		0. 821		0.335		0.912	
	**Estimate**^**†**^	**P value**	**Estimate**	**P value**	**Estimate**	**P value**	**Estimate**	**P value**	**Estimate**	**P value**
**Group1 T2-T1**	-0.39	0.001	-0.40	0.011	-0.49	0.002	-0.21	0.371	-0.38	0.005
**T3-T1**	-0.40	0.001	-0.59	0.001	-0.41	0.022	-0.09	0.700	-0.51	0.003
**Group 0 T2-T1**	-0.73	0.009	-0.48	0.118	-0.40	0.152	-0.78	0.002	-0.42	0.127
**T3-T1**	-0.69	0.013	-0.08	0.783	-0.39	0.174	-0.70	0.038	-0. 27	0.352
**BPS/P value at baseline**	0.999		0.432		0.594		0.616		0.083	
**Group1 T2-T1**	-2.14	0.240	-3.75	0.113	-4.64	0.047	-3.60	0.181	-235	0.241
**T3-T1**	-4.19	0.031	--1.97	0.460	-5.00	0.067	-6.64	0.026	-2.48	0.305
**Group 0 T2-T1**	-4.59	0.124	-2.26	0.508	-5.62	0.059	-6.29	0.071	-7.16	0.011
**T3-T1**	-0.38	0.901	-1.16	0.745	-2.37	0.466	-5.34	1.35	-4.40	0.161
**BPD/P value at baseline**	0.989		0.091		0.520		0.445		0.665	
	Total Resistance		despair		Difficulty		Therapy		Emotions	
**Group1 T2-T1**	-1.40	0.185	-2.26	0.088	-2.69	0.037	-3.11	0.054	-2.20	0.049
**T3-T1**	-4.30	<0.001	-5.18	0.001	-5.81	<0.001	-5.16	0.004	-3.85	0.005
**Group 0 T2-T1**	-2.33	0.193	0.12	0.950	-1.58	0.367	-0.06	0.974	-2.31	0.174
**T3-T1**	-3.19	0.090	-1.27	0.539	-2.49	0.193	-3.94	0.068	-3.81	0.041
**BMI/P value at baseline**	0.784		0.046		0.345		0.206		0.508	
**Group1 T2-T1**	0.28	0.416	-0.43	0.315	0.89	0.073	1.20	0.144	0.44	0.262
**T3-T1**	-0.44	0.215	-0.76	0.112	-0.91	0.113	-0.82	0.279	-0.79	0.099
**Group 0 T2-T1**	-0.49	0.605	0.54	0.599	-1.16	0.299	-1.50	0.184	-0.65	0.512
**T3-T1**	-0.76	0.431	-1.04	0.319	-1.39	0.168	1.42	0.224	-0.93	0.368
**HDL/P value at baseline**	0.415		0.241		0.790		0.769		0.045	
**Group1 T2-T1**	-0.51	0.439	0.59	0.520	-0.50	0.572	-1.90	0.106	-0.58	0.441
**T3-T1**	-1.13	0.100	0.61	0.560	0.37	0.740	-1.49	0.270	0.24	0.797
**Group 0 T2-T1**	1.21	0.548	-2.05	0.369	0.82	0.702	1.10	0.631	3.15	0.135
**Continue:**	**Total resistance**		**despair**		**Difficulty**		**Therapy**		**Emotions**	
**T3-T1**	1.14	0.570	-2.51	0.283	0.57	0.749	0.76	0.739	2.27	0.296
	Total resistance		despair		Difficulty		Therapy		Emotions	
**LDL/P value at baseline**	0.699		0.235		0.322		0.975		0.267	
**Group1 T2-T1**	-2.58	0.451	-5.35	0.210	-4.34	0.557	-7.47	0.144	-4.25	0.271
**T3-T1**	-0.50	0.889	1.43	0.766	2.97	0.557	2.26	0.672	-0.15	0.973
**Group 0 T2-T1**	-3.66	0.539	-6.75	0.298	5.31	0.356	-1.52	0.818	4.04	0.481
**T3-T1**	-3.17	0.594	-6.35	0.357	4.37	0.479	-3.54	0.592	2.43	0.703
**TG/P value at baseline**	0.564		0.740		0.095		0.791		0.535	
**Group1 T2-T1**	-3.63	0.602	-1.90	0.840	-17.79	0.068	2.99	0.777	-1.00	0.901
**T3-T1**	9.90	0.169	23.53	0.042	6.67	0.541	4.85	0.645	14.16	0.154
**Group 0 T2-T1**	17.29	0.303	12.42	0.462	37.2	0.049	-5.49	0.733	12.68	0.449
**T3-T1**	18.50	0.271	15.21	0.390	38.4	0.050	8.23	0.609	26.52	0.135

Group 1: achieved reduction at T2 (delta between T2 and T1 <0); group 0: those who failed to achieve reduction (delta between T2 and T1 = >0).

† Estimate = delta between time points ^α^P Value for differences between groups at baseline.

Models for Emotions were adjusted for age; models for difficulty were adjusted for family status; models for despair were adjusted for education and family status.

## Discussion

Our main findings suggest that patients with type 2 diabetes who participated in TPE groups and succeeded in reducing their resistance to treatment achieved better control of the disease which was sustained for at least one year of follow up. The reduction obtained was not only statistically but clinically significant in most cases.

Participants, whose resistance to treatment was significantly reduced, were those who began the intervention with higher levels of resistance. This indicates a regression to the mean effect, or that the TPE was actually more effective for those who have had more difficulties coping with diabetes. Thus, at this point we may conclude that for TPE, it would be more efficient to recruit the less motivated patients who are coping less well.

Glycosylated Hemoglobin (HbA1C) was the most significant clinical measure that was reduced in alignment with reduction in resistance to treatment. This may indicate that overcoming resistance to treatment is crucial for better management of diabetes and that the achieved behavior changes influenced adherence to medication and dietary guidelines. On the other hand, blood pressure was reduced but not consistently significant among the resistance cores, and for other clinical measurements: cholesterol; triglycerides and BMI no significant reduction was observed in both study groups. There is no clear cut explanation for this, but it is possible that it is because diabetes was the focus of the TPE and the agenda emphasizes diabetes management. We analyzed these variables to allocate an indirect effect.

Resistance to treatment was suggested by previous studies, as an important factor in the success of the lifestyle modifications and in the achievement of desired outcomes [14,15], mainly in patients with no clear-cut symptoms, as is the case in most patients diagnosed with diabetes. The resistance of people with diabetes to treatment and its reasons are poorly understood. The patient often understands the need for treatment or for lifestyle change and even intends to make changes to their lifestyle, but in practice does not take the necessary actions to bring about these changes. This may be partly explained by theoretical models, such as the Trans Theoretical Model (TTM), which emphasizes verbal and behavioral processes that are associated with four stages of change: thinking about change, making a decision to change; active change and maintenance, in addition to patients’ self-efficacy and belief in their ability to make that change [7-9].

The question is what are the psychological factors influencing adherence to treatment. Self-efficacy and low levels of resistance to treatment have been well documented as having an impact on adherence to treatment and therefore play a role in the clinical outcome. Moreover, it was suggested that Self-efficacy measurement could play an important role in diabetes management, in order to identify where the patient is most likely to adhere to recommended self-care treatment [13][16]. We did not measure self efficacy, because a robust and negative correlation was found between self-efficacy and resistance to treatment (the higher the resistance to treatment, the less confident the patient is in his/her ability to manage the disease) [16]. Although it is possible that measuring self-efficacy would have added some information regarding the patients’ state of mind before and after the intervention, we believe that the resistance to treatment was a good surrogate for self efficacy, and adding more items to the questionnaire would have reduced our response level.

In our study not all four resistance core components were equally associated with improvement in the clinical biochemical outcomes. The reduction in HbA1C was associated with reduction in feelings of despair, difficulties and emotions and the reduction in blood pressure was associated mainly with difficulty and emotions. It is possible that reducing resistance to treatment at an emotional level, enhances acceptance of both diabetes and hypertension as a chronic asymptomatic disease, and once the emotional barrier is overcome the actual changes evolve. In comparison, weight loss, for example, is associated with a different type of acceptance which requires a different set of lifestyle changes.

Regarding the statistical techniques- as mentioned earlier we had an unbalanced design of repeated measurements; we used linear mixed models for each dependent variable separately as this is the most appropriate approach for this type of design.

Some limitations of this study should be addressed. This study is a program review, evaluating changes in outcomes before and after the intervention with no control group. The study subjects were not randomly assigned. Only those who agreed to take part in the evaluated process participated and were followed up to T3. This probably leads to selection bias. However, when an intervention is an integral part of clinical care there is no ethical choice but to accept those who agreed to participate, both in the intervention and the evaluation process and to adjust in the statistical analysis for the personal variables, as we did. It is also realistic to expect that in the future, patients with diabetes who choose to participate in TPE groups, will self-select in a manner similar to that presented here, making the findings more relevant in the “real world”. Furthermore, as this study did not include a control group, we can not assume a causal effect by the intervention on the resistance components and the clinical measurements. It is therefore recommended that future research focuses on randomized controlled interventions, with in-depth examination of the content of diabetes education groups, and its impact on behavioral and clinical outcomes controlling for other important variables such as duration of diabetes, depression, type of diabetes medication etc.

## Conclusions

The findings from this study support the use of group education for diabetes self-management. The RTQ appears to be a useful tool for the measurement of diabetes related behavioral interventions. Further research is essential to better understand these associations.

## Competing interests

We declare hereby that in the past five years we did not received any reimbursements, fees, funding, or salary from an organization that may in any way gain or lose financially from the publication of this manuscript, either now or in the future.

We hereby declare that none of us hold any stocks or shares in an organization that may in any way gain or lose financially from the publication of this manuscript, either now or in the future.

We hereby declare that none of us hold or are currently applying for any patents relating to the content of the manuscript.

We hereby declare that none of us received reimbursements, fees, funding, or salary from an organization that holds or has applied for patents relating to the content of the manuscript.

We hereby declare that none of us has any other financial competing interests.

We hereby declare that none of us has any non-financial competing interests (political, personal, religious, ideological, academic, intellectual, commercial or any other) in relation to this manuscript.

This study was funded by a grant from The Maccabi Institute for Health Services Research.

## Authors’ contributions

LV participated in the study design and data analysis and supervised the intervention. RE participated in the study design and in the sequence alignment of the intervention. RP and KD administered the questionnaires and coordinated the study. MM participated in the study design and adapted the questionnaires .ADH consulted and supervised the study and helped to draft the manuscript .All authors read and approved the final manuscript.

## Pre-publication history

The pre-publication history for this paper can be accessed here:

http://www.biomedcentral.com/1472-6823/13/61/prepub
